# Pretransplant Treatment to Avoid Recurrent Membranous Nephropathy in a Kidney Transplant Recipient: A Case Report

**DOI:** 10.1016/j.xkme.2024.100822

**Published:** 2024-04-12

**Authors:** Erik L. Lum, Jonathan E. Zuckerman, Lama Abdelnour, Jennifer Terenzini, Gurbir Singh, Suphamai Bunnapradist

**Affiliations:** 1 Division of Nephrology, Department of Medicine, UCLA David Geffen School of Medicine, Los Angeles, California; 2 Department of Pathology and Laboratory Medicine, UCLA David Geffen School of Medicine, Los Angeles, California; 3 Department of Transplant Services, Kidney and Pancreas Transplant, UCLA, Los Angeles, California

**Keywords:** Kidney transplant, membranous nephropathy, recurrent glomerulonephritis

## Abstract

Kidney transplant candidates with high anti–M-type phospholipase A2 receptor antibody activity may be at increased risk for early postkidney transplant recurrence and allograft loss. Pretransplant treatment to induce serological remission may be warranted to improve allograft survival. In this case report, a patient seeking their third kidney transplant, who lost 2 prior living donor transplants from early recurrent membranous nephropathy, underwent pretransplant treatment for membranous nephropathy with serological remission and no evidence of recurrent disease.

## Introduction

Recurrence of primary glomerulonephritis after kidney transplantation may result in premature graft failure. Anti–M-type phospholipase A_2_ receptor (PLA_2_R) antibody has been identified as the causative antibody in over 75% of primary membranous nephropathy (MN) cases. Antibody levels are associated with disease activity. Reported postkidney transplant recurrence of MN varies from 10%-40%. It is hypothesized that the presence of anti-PLA_2_R antibodies at the time of kidney transplant increases the risk of recurrent MN. Herein we present a case report of a patient who lost 2 prior kidney transplants from early recurrent MN who received pretransplant therapy to lower anti-PLA_2_R antibody levels before undergoing a successful third kidney transplant.

## Case Report

A man in his 50s with kidney failure from MN presented for consideration of his third kidney transplant. He initially presented in his 20s with acute onset anasarca and on laboratory testing was found to have an elevated creatinine and nephrotic range proteinuria. He underwent a native kidney biopsy showing MN and significant scarring. Treatment at the time was deferred due to the advanced nature of his kidney disease, and he eventually started dialysis 3 years later. He was on hemodialysis for 3 years before receiving a living-unrelated kidney transplant. His initial course was uncomplicated. He developed nephrotic range proteinuria approximately 1 year after kidney transplantation with progressive glomerular filtration rate decline and allograft failure 4 years posttransplant.

He returned to hemodialysis for 1 year before receiving a 2-haplotype matched kidney from his sister. Within 1 month of transplantation, he developed nephrotic range proteinuria with 14.4 g. He underwent a kidney transplant biopsy showing MN in anti-PLA_2_R staining. He underwent treatment with 2 doses of rituximab and 6 months of corticotropin therapy. His proteinuria improved to 1.1 g. After cessation of corticotropin therapy, his proteinuria recurred and his glomerular filtration rate declined, resulting in resuming dialysis 3 years after receiving his second kidney transplant.

He returned for consideration of a third kidney transplant after having been on hemodialysis for 7 years. He had been declined by several other local transplant centers due to concerns for high risk for recurrence and allograft loss and was referred to our center. Anti-PLA_2_R antibody testing was positive at a level of 79 relative units [RU]/mL. He was also sensitized from his prior transplants with a calculated panel reactive antibody of 100% and 3 donor-specific HLA antibodies to his potential donor.

**Figure 1:**
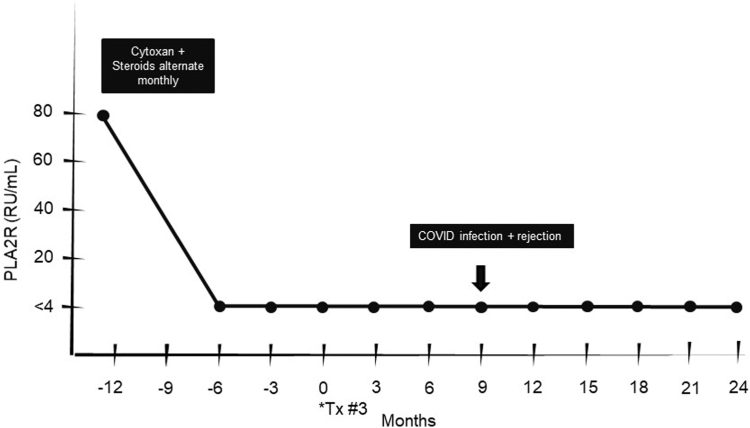
Key clinical time points. Patient time course around kidney transplant #3. Treatment with cytoxan and steroids, alternating monthly, performed 12 months before receiving kidney transplant. Anti-PLA_2_R levels measured before treatment, following treatment, and at 3-month intervals are shown. Abbreviations: COVID-19, coronavirus disease 2019; PLA_2_R, M-type phospholipase A2 receptor; Tx, transplant.

After extensive counseling, he received pretransplant treatment for MN with the goal of achieving a low anti-PLA_2_R level and proceeding with living donor kidney transplantation through paired exchange (Fig 1). He received cyclophosphamide 1 mg/kg daily, reduced for dialysis, during months 1, 3, and 5, and prednisone 0.5 mg/kg in months 2, 4, and 6. Anti-PLA_2_R titers were measured after therapy completion and decreased to <4 RU/mL by month 6 and remained at that level in the 6 months necessary to locate a compatible kidney donor through the paired exchange.

**Figure 2:**
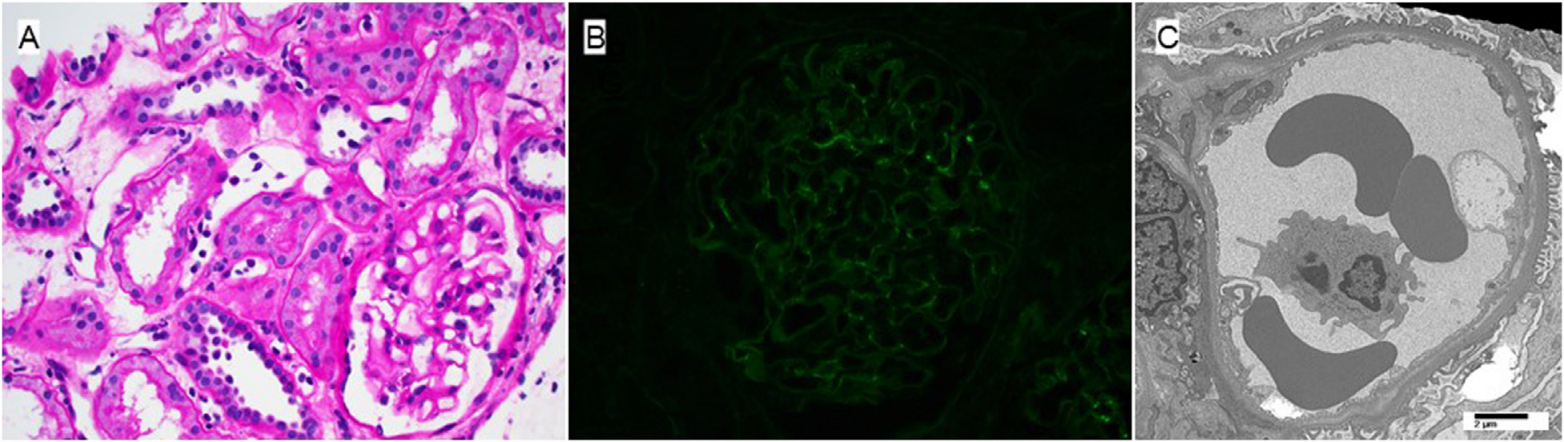
(A) Light micrograph demonstrating peritubular capillaritis (periodic acid–Schiff stain; 400×). (B) Immunofluorescence staining for IgG demonstrating segmental granular capillary wall deposits (400×). (C) Electron micrograph demonstrating chronic transplant glomerulopathy.

A potential living donor in their 20s was identified for which a single donor-specific antibody to A68:01 with low-level mean fluorescence index was present. The recipient underwent desensitization therapy with rituximab and intravenous immunoglobulin and underwent successful kidney transplantation with antithymocyte globulin induction. After the kidney transplant, he achieved a baseline creatinine level between 1.4 and 1.5 mg/dL, and serial testing revealed no evidence of proteinuria and persistent anti-PLA_2_R levels <4 RU/mL on testing every 3 months posttransplant. He was maintained on prednisone, tacrolimus, and mycophenolate mofetil. One year posttransplant, he developed coronavirus disease 2019 pneumonia, and his mycophenolate mofetil was held. He received remdesivir and presented 2 weeks later with an elevated creatinine level. A kidney biopsy (Fig 2) showed acute antibody-mediated rejection with only minimal IgG deposition seen by immunofluorescence microscopy without other light or electron microscopic features of no significant features of MN. A PLA_2_R immunofluorescence stain performed on the biopsy specimen was negative. Single antigen testing showed no donor-specific antibody. He underwent therapy with plasmapheresis for 5 sessions and received postpheresis intravenous immunoglobulin with the return of creatinine level to baseline. He is now over 2 years postkidney transplantation and continues to exhibit stable, excellent allograft function with no evidence of recurrent MN and negative for anti-PLA_2_R antibodies.

## Discussion

Effective pretransplant treatment for MN with serological remission prevented recurrent disease and allowed this patient to avoid early allograft loss. Recurrent glomerulonephritis postkidney transplant is unpredictable and a common cause of late graft loss.^1^ In an analysis of the Australia and New Zealand Dialysis and Transplant Registry, recurrence of primary MN occurred in approximately 20% of recipients, was the cause of allograft loss in over 60% of patients, and was associated with a >3-fold increase in death-censored graft failure.^2^ This case demonstrates the significant burden of recurrent MN in a transplant recipient without serological remission. In addition to his high risk for recurrence, he was very highly sensitized from his prior kidney transplants.

The discovery of anti–M-type PLA_2_R antibody as a causative antibody for primary MN (PMN) has dramatically altered the landscape of PMN diagnosis and management.^3^ It is now believed that PMN develops from circulating autoantibodies against antigens on the podocyte surface, with several other antibodies recently identified, even in secondary causes.^4^ The high specificity of anti-PLA_2_R antibodies for membranous disease has led to proposals that kidney biopsy may be deferred in patients with nephrotic syndrome, preserved kidney function with an estimate glomerular filtration rate >60 mL/min/1.73 m^2^, and positive for anti-PLA_2_R antibodies. If a biopsy is performed, histological staining for anti-PLA_2_R antibodies reveals a characteristic intense staining along the glomerular capillary wall.^5^ In our patient, his initial diagnosis and first kidney transplant occurred before anti-PLA_2_R antibody testing and histological staining availability. His second transplant testing for anti-PLA_2_R antibodies was available at only a few centers, and his biopsy samples were sent to another institution. Serum testing was not available at the time. The presence within 1 month of transplantation suggests that his early recurrence was due to circulating antibodies before transplantation.

Antibody levels are useful in PMN to determine disease activity and response to therapy. Low-level titers are associated with spontaneous remission, whereas high levels are associated with progressive disease. Antibody levels appear several months before the clinical manifestations of PMN.^6^ These characteristics make serological anti-PLA_2_R testing an attractive option for managing patients with PMN and in assessing the recurrence of disease posttransplantation.^7^ Pooled results of 2 studies evaluating anti-PLA_2_R seropositivity before transplantation showed a cutoff titer above 29 RU/mL was predictive of recurrence with a sensitivity of 85% and specificity of 92%.^8^^,^^9^ The following testing in our patient in his second transplant may have been useful in determining if serological remission occurred and if treatment could be stopped or resumed if anti-PLA_2_R antibody levels returned. Achieving and maintaining remission before transplant resulted in no evidence of recurrence in our recipient.

Two tests for anti-PLA_2_R antibodies are commercially available currently. The enzyme-linked immunosorbent assay (Euroimmun) is the most used assay and allows quantitative assessment. A cell-based indirect immunofluorescence assay is more sensitive for detecting low levels of anti-PLA_2_R but does not allow for quantitative assessment. In our case, given the use of pretransplant alkylating treatment and the need for serological remission to permit and time his paired exchange kidney transplantation, enzyme-linked immunosorbent assay was used.

Currently, the goal of therapy in PMN is to induce serological remission and in those with serological remission, to control proteinuria with medical, rather than immunosuppressive, therapy. Historically the Ponticelli regimen using alternating alkylating agents and steroids has been the mainstay of therapy.^10^ Because the discovery that PMN is an autoimmune disease, addressing antibodies with the less toxic B cell-depleting agent rituximab has garnered attention. In the seminal MENTOR trial, rituximab was superior at achieving remission compared to cyclosporine-treated patients, 60% vs 20%.^11^ However, cyclosporine is considered inferior to alkylating therapy and as a result, 2 trials comparing cyclophosphamide and rituximab were performed. In the STARMEN trial, cyclophosphamide therapy was superior to a combination of tacrolimus and rituximab in producing complete remission by 24 months, 60% vs 26%.^12^ The main concern with this trial was that rituximab was given 6 months after calcineurin inhibitor treatment and at a low dose of 1 g and may not represent a true head-to-head comparison. Anti-PLA_2_R antibody decline lagged during tacrolimus treatment in the tacrolimus/rituximab arm. In 2021, the RI-CYCLO trial directly comparing rituximab and cyclophosphamide showed comparable remission at 24 and 36 months.^13^ Our patient was treated before the RI-CYCLO results being published. Given these results, it would be reasonable to treat with rituximab and reserve alkylating therapy in a kidney transplant candidate requiring pretransplant MN therapy for rituximab failure, especially if rituximab is already being considered for HLA desensitization. Our patient received rituximab following remission and for the purpose of HLA desensitization, but this may have contributed to sustained serological remission.

Achieving serological remission with pretransplant treatment of PMN in transplant candidates with high levels of anti-PLA_2_R antibodies can reduce the risk of posttransplant recurrence.
